# Maladie de Still de l'adulte compliquée d'une hépatite fulminante

**DOI:** 10.11604/pamj.2015.20.446.5935

**Published:** 2015-04-30

**Authors:** Madiha Mahfoudhi, Rafik Shimi

**Affiliations:** 1 Service de Médecine interne A, Hôpital Charles Nicolle, Tunis, Tunisie

**Keywords:** Maladie de Still, hépatite fulminante, éruption cutanée, Still disease, fulminant hepatitis, skin rash

## Image en medicine

La maladie de Still de l'adulte (MSA) est une maladie inflammatoire rare d’étiopathogénie mal élucidée qui se manifeste par une fièvre associée à des arthrites, une éruption cutanée fugace et des atteintes multi-viscérales. Elle se complique rarement d'une hépatite fulminante qui aggrave le pronostic. Patiente âgée de 38 ans admise pour une fièvre prolongée associée à des arthralgies de type inflammatoire de coudes et des genoux. Elle présentait une éruption maculo-papuleuse non prurigineuse érythémateuse des jambes, des cuisses et des avant bras fugace coïncidant avec les pics fébriles. L'examen biologique a révélé un syndrome inflammatoire biologique, une hyper férritinémie à 3 fois la normale, une hyperleucocytose à 13200/mm3. Par ailleurs, elle avait une cytolyse hépatique à 5 fois la normale et des signes d'insuffisance hépatique. Le diagnostic de MSA a été évoqué après avoir éliminé une cause infectieuse, une connectivite, une vascularite, une hémopathie ou un néoplasie solide. Selon la classification de Yamaguchi, notre patiente avait 4 critères majeurs (arthralgies, fièvre, éruption cutanée, hyperleucocytose avec polynucléaires neutrophiles > 80%) et 2 critères mineurs (Perturbations du bilan biologique hépatique et Absence d'anticorps antinucléaires et de facteur rhumatoïde). Elle a été mise sous corticothérapie générale. L’évolution était marquée par la disparition de tous les signes cliniques et biologiques. Un traitement à base de Méthotrexate lui a été par la suite instauré comme traitement de fond. Elle n'a pas présenté de rechute de sa maladie avec un recul de 3 ans.

**Figure 1:**
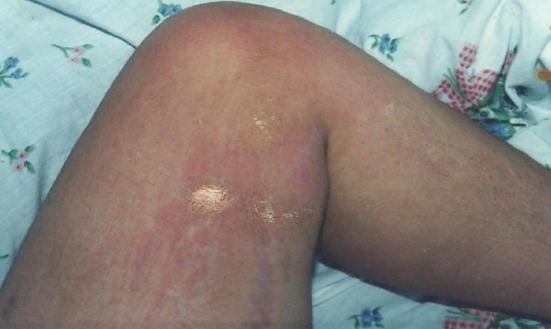
Eruption cutanée rose saumon au niveau de la face interne de la cuisse gauche associée à des lésions de grattage

